# The overlooked role of manganese in biodegradation studies of higher aminopolyphosphonates

**DOI:** 10.1007/s11356-025-37105-9

**Published:** 2025-11-04

**Authors:** Kleanthi Kourtaki, Philipp R. Martin, Stefan B. Haderlein

**Affiliations:** 1 Department of Geosciences, Eberhard Karls Universität Tübingen, Tübingen, Germany; 2 Division for Environmental Geosciences, Centre for Microbiology and Environmental Systems Science, University of Vienna, Vienna, Austria

**Keywords:** Synthetic phosphonates, Complexing agents, IDMP, P-cycling, APP degradation, *Achromobacter*

## Abstract

Higher aminopolyphosphonates (APPs, with three or more C–P bonds) are common complexing agents, but their environmental fate and degradation/transformation pathways remain poorly understood. While abiotic degradation of higher APPs has been reported, recent studies have also proposed the potential for enzymatic breakdown based on laboratory model systems. However, herein, we show rapid transformation of two representative higher APPs – aminotris(methylenephosphonate) (ATMP, with three C–P bonds) and ethylenediaminetetramethylenephosphonate (EDTMP, with four C–P bonds) – in bacterial growth media both in the presence and the absence of *Achromobacter insolitus* strain Kg 19. The abiotic transformation was driven by trace levels of dissolved Mn(II) – typically present in mineral solutions used in growth media. Mn(II) catalyzed the abiotic oxidation of ATMP by molecular oxygen, yielding iminodimethylenephosphonate (IDMP) and orthophosphate (*o*-PO₄) as transformation products, via a known mechanism previously described in simpler matrices. The addition of ethylenediaminetetraacetic acid (EDTA) as metal complexing agent retarded but did not prevent the abiotic transformation. Bacterial growth under phosphorous (P)-limiting conditions in the presence of ATMP and EDTMP was thus supported by the formation of P-containing transformation products of the concurrent abiotic degradation rather than biotransformation of ATMP and EDTMP. These findings reveal a critical limitation in prior biodegradation studies of higher APPs, which likely overestimated biotransformation by overlooking Mn(II)–mediated abiotic degradation. Future research must redesign biodegradation approaches and disentangle biotic and abiotic mechanisms to accurately evaluate the actual biodegradability of higher APPs.

**Graphical Abstract:**

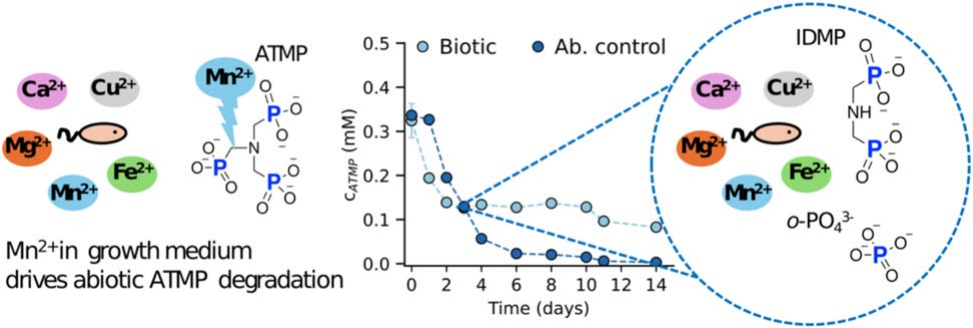

**Supplementary Information:**

The online version contains supplementary material available at 10.1007/s11356-025-37105-9.

## Introduction

Higher aminopolyphosphonates (APPs) are synthetic organophosphorus compounds featured with the presence of one or more amine (–NH_2_) and three or more phosphonic acid groups attached to carbon atoms (–P(= O)(OH)_2_) (Studnik et al. 2015). Their market introduction was driven by a need for more effective and environmentally acceptable chelating agents as alternatives to traditional polyphosphates and polycarboxylates like ethylenediaminetetraacetate (EDTA) (Knepper 2003; Jaworska et al. 2002). APPs are particularly effective in hard waters. The fact that they are effective at concentrations one or more magnitudes lower than polyphosphates further contributes to their widespread use and commercial importance (Studnik et al. 2015). Their strong metal-chelating properties and ability to inhibit scale formation have made them popular in industrial and household applications such as water treatment, detergents, and metal cleaning (Knepper 2003).

APPs primarily enter the environment through wastewater discharge or point sources like cooling system effluents (Fischer 1993; Jaworska et al. 2002). Although generally regarded as non-toxic to aquatic biota such as invertebrates and fish (Knepper 2003; HERA 2004), they can have indirect effects by binding essential trace metals, reducing their bioavailability, and indirectly compromising growth by altering nutrient dynamics (Schowanek et al. 1996). As a result, their release into aquatic environments can disrupt ecosystems, affecting microbial competition for these essential minerals. Recently, it was shown that glyphosate is a transformation product (TP) of diethylenetriamine penta(methylene phosphonate) (DTPMP) during manganese-catalyzed oxidation, as well as during incubation in sewage sludge (Röhnelt et al. 2025b; Engelbart and Bieger et al., 2025).

Various abiotic degradation processes, such as photolysis, ozonation, or Mn(II)-driven oxidation by molecular oxygen, have been reported to efficiently break them down (Greenlee et al. 2014; Lesueur et al. 2005; Kuhn et al. 2018; Martin et al 2022; Marks et al. 2023; Röhnelt et al. 2023). APPs were previously considered largely resistant to degradation due to their strong C–P bond (Ternan et al. 1998). To date, sorption to sewage sludge is regarded as the primary cause for their partial retention in waste water treatment plants (WWTPs) (Fischer 1993; Nowack 1998, 2002; Knepper 2003). Biodegradation in WWTPs was initially considered negligible due to the high availability of inorganic phosphorus (P) which inhibits the induction of enzymes like C–P lyases – key enzymes for breaking down synthetic APPs under P-limiting conditions (McGrath et al. 1997; Kononova and Nesmeyanova 2002; Santos-Beneit 2015; Stosiek and Klimek-ochab 2020). These observations are in line with the outcomes of standard OECD biodegradability tests, which have consistently shown low or negligible levels of biodegradation for compounds such as aminotris(methylenephosphonate (ATMP) (HERA 2004). The poor performance of APPs in these tests is not necessarily due to inherent environmental persistence but rather reflects test conditions that fail to promote the activity of specialized phosphonate-degrading microorganisms. In particular, the presence of excess phosphate buffers in test media inhibits the microbial pathways responsible for APP breakdown, mirroring the limitations observed under WWTP conditions (Strotmann et al. 2023).

Nevertheless, recent studies proposed biodegradation of higher APPs. Cyanobacterial strains such as *Spirulina platensis*, *Arthrospira fusiformis*, and *Anabaena variabilis* were shown to sustain growth by metabolizing hexamethylenediamine-N,N,N′,N′-tetrakis(methylphosphonate) or DTPMP (Forlani et al. 2011). Bacterial strains have also been isolated with demonstrated APP-degrading abilities. *Ochrobactrum* sp. strain BTU1 (abbreviated as *O*. BTU1), for instance, was recently enriched from a DTPMP solution, and degradation tests with EDTMP showed 80% decrease in ethylenediaminetetramethylenephosphonate (EDTMP) concentration (*c*_0_ = 0.23 mM), alongside biomass production. In subsequent tests, a decrease in ATMP concentration was also shown when provided as P-source to *O*. BTU1 and when sewage sludge was used as inoculum (Riedel et al. 2023). In an earlier study, Schowanek and Verstraete (1990) showed that EDTMP was degraded by activated sludge cultures, achieving up to 85% removal. Further screening identified enrichment cultures capable of utilizing higher APPs as sole C, N, or P sources; however, analytical limitations restricted this study to phosphate (PO₄^3−^) quantification rather than direct measurement of parent compounds (McGrath et al. 1997). PO_4_^3−^ has often been used as proxy for APP transformation (Schowanek and Verstraete 1990; Riedel et al. 2024). Recent evidence, however, suggests that this approach may overestimate actual degradation, as PO₄^3^⁻ quantification can be skewed by matrix interferences in samples containing high levels of higher APPs (Guo et al. 2025). Thus, relying solely on PO₄^3^⁻ release is insufficient for accurately assessing higher APP transformation.

Our study critically evaluates earlier studies on higher-APP biodegradability by investigating the transformation of ATMP and EDTMP – by *Achromobacter insolitus* strain Kg 19 under P-limiting conditions using commonly applied culture media. We critically evaluate the influence of media composition – particularly the presence of trace metals – on the degradation behavior of these analytes. ATMP and EDTMP were chosen due to their widespread use in cleaning products across Europe (HERA 2004; Jaworska et al. 2002) and their detection in WWTP effluents (Rott et al. 2018). Experiments were conducted in C- and N-rich media supplemented with bivalent cations, following standard protocols for assessing phosphonate biodegradability. By examining both microbial and media-related factors on higher-APP transformation, this work aims to advance our understanding of the fate of higher APPs and inform future risk assessments and treatment strategies.

## Materials and methods

### Chemicals and materials

EDTMP (> 96.0%) has been purchased in solid form from Zschimmer and Schwarz (Lahnstein, Germany. ATMP (> 97.0%) and IDMP (≥ 97%) have been purchased as solids from Sigma Aldrich (Steinheim, Germany). MOPS buffer (99.5%) was bought from Carl Roth (Karlsruhe, Germany) and sodium acetate (> 99.5%) from Chemsolute (Renningen, Germany). The cation-exchange resin (DOWEX 50 W × 8, 100–200 mesh, H^+^-form) was purchased from Roth (Karlsruhe, Germany). For eluent preparation, sodium hydroxide (NaOH, 49–51%) was used (Supelco, Merck, Darmstadt, Germany). Additional chemicals used for the experiments were purchased from Merck in their highest purity (Darmstadt, Germany). Ultrapure water (*ρ* > 18MΩ cm) was used for all experiments obtained by a Barnstead GenPure system (Thermo Fischer Scientific, Germany).

### Setup of biodegradation batch experiments

Biotransformation experiments were conducted in 100-mL glass serum bottles, continuously shaken at 150 rpm on a rotary shaker at room temperature (21 ± 1 °C) under ambient light conditions, as previously described by Kourtaki et al. (2025). The bacterial strain *Achromobacter insolitus* strain Kg 19 (hereafter referred to as *A*. Kg 19), originally isolated from the Krasnodar region in Russia (45° 03′ 10.8″ N, 38° 52′ 22.8″ E), was obtained as freeze-dried culture from the Russian Centre of Microorganisms (VKM) (VKM B-3295) (Tarlachkov et al. 2020). *A*. Kg 19 was cultivated in Medium_1_, buffered at pH 7.0 ± 0.1 with 50 mM MOPS. The medium included 10 g/L Na-glutamate, 2 g/L NH_4_Cl as N-source, 0.5 g/L K_2_SO_4_, 0.2 g/L MgSO_4_ × 7 H_2_O, 1 mL/L of a 7-vitamin solution, and a trace element solution (TES) with: 20 mg/L ZnSO_4_ × 7H_2_O, 10 mg/L CaCl_2_ × 6 H_2_O, 2.5 mg/L FeSO_4_ × 7 H_2_0, 2 mg/L CuSO_4_ × 5 H_2_O, 1 mg/L MnSO_4_ × H_2_O, 0.3 mg/L Na_2_MoO_4_, 0.06 mg/L H_3_BO_3_, and 0.05 mg/L NiCl_2_ × 6H_2_O. Details on the preparation of bacterial cultures are provided in the Supporting Information.

Each experiment consisted of three biotic replicates and three abiotic controls to assess potential interactions with the APPs and the medium.

### Setup of abiotic transformation batch experiments

The effect of different medium components on ATMP was tested by preparing 0.5 mM of ATMP in seven solutions as outlined in Table 1. (1) 50 mM MOPS, (2) 50 mM MOPS with 1 mL/L of the 7-vitamin solution, (3) 50 mM MOPS with TES, (4) same as (3) with 100 µM Na_2_-EDTA × 2H_2_O, (5) sterile Medium_1_ (as described in “Setup of biodegradation batch experiments” subsection in Materials and methods section), (6) same as (5) with 100 µM Na_2_-EDTA × 2H_2_O, and (7) sterile Medium_2_ used for cultivation of *Ochrobactrum pituitosum* strain GPr1-13 (abbreviated as *O*. GPr1-13) in previous research. Medium_2_ consisted of 10 g/L Na-glutamate, 5 g/L NH_4_Cl as N-source, 0.5 g/L K_2_SO_4_, 0.16 g/L MgSO_4_ × 7H_2_O, 0.08 g/L CaCl_2_ × 6H_2_O, 1 mL/L of a 7-vitamin solution and was buffered at pH 7.0 ± 0.1 with 50 mM MOPS. The effect of Mn(II) was tested by examining three different concentrations (2.5, 6, and 10 µM) using MnSO_4_ × 7H_2_0 prepared in ultrapure water. The experiments were conducted in sterile 50-mL centrifugation tubes (polypropylene, Fisher Scientific, Waltham, MA, USA) placed in a rotary shaker running at 25 rpm.

**Table 1:** Experimental setup testing the effect of seven different medium components, each with an initial ATMP concentration of 0.5 mM

Setups	MOPS	Vitamins	TES	EDTA	Medium_1_	Medium_2_
1	✓					
2	✓	✓				
3	✓		✓			
4	✓		✓	✓		
5	✓	✓	✓		✓	
6	✓	✓	✓	✓	✓	
7	✓	✓				✓

All transformation experiments were conducted in triplicates at room temperature (21 ± 1 °C). ATMP and EDTMP stock solutions (*c* = 25 mM) were prepared in ultrapure water, adjusted at pH = 7.0 ± 0.1 using 1 M NaOH, and filtered-sterilized through 0.2-µm syringe filters ((surfactant-free) cellulose acetate, Sartorius, Göttingen, Germany).

### Sampling of transformation batch experiments

For each sampling point, 1 mL of sample aliquot was withdrawn under sterile conditions and added to a 2-mL centrifugation tube containing 50 mg ± 2 mg of cation exchange resin to quench any potential interaction between APPs and cations present in the solutions tested. The vials were placed for 1 h on an overhead shaker. Afterward, the resin-free supernatant containing the APPs in their free (uncomplexed) form was stored at − 20 °C in the dark till concentration analysis, if not analyzed directly. The treatment and storage procedure had no adverse effect with respect to the stability of the APPs. Samples containing cells were first centrifuged (10 min at 12,000 rcf), and the biomass-free supernatant was subsequently treated with the cation exchange resin.

### ATMP, EDTMP, and IDMP quantification

Quantification of ATMP, EDTMP, and IDMP was conducted by ion chromatography (Metrohm, Herisau, Switzerland) coupled to integrated amperometric detection (Wall- Jet Cell, Metrohm) (Röhnelt et al. 2025a, b). Samples treated with cation-exchange were diluted 1:25 with ultrapure water for analysis. Calibration was carried out using external standards ranging from 0.01 to 20 µM, and normalization was performed based on repeated injections of control standards.

### Orthophosphate (*o*-PO_4_) quantification

Orthophosphate (*o*-PO_4_) formation as a transformation product of APP degradation was monitored photometrically at 710 nm with a UV5Bio spectrophotometer (Mettler Toledo, Germany) using the molybdenum-blue method adapted from Murphy and Riley (1962) after a 10 × dilution of the samples in a 1-cm PP cuvette.

## Results and discussion

### ATMP and EDTMP transformation in inoculated and sterile growth medium

**Fig. 1:**
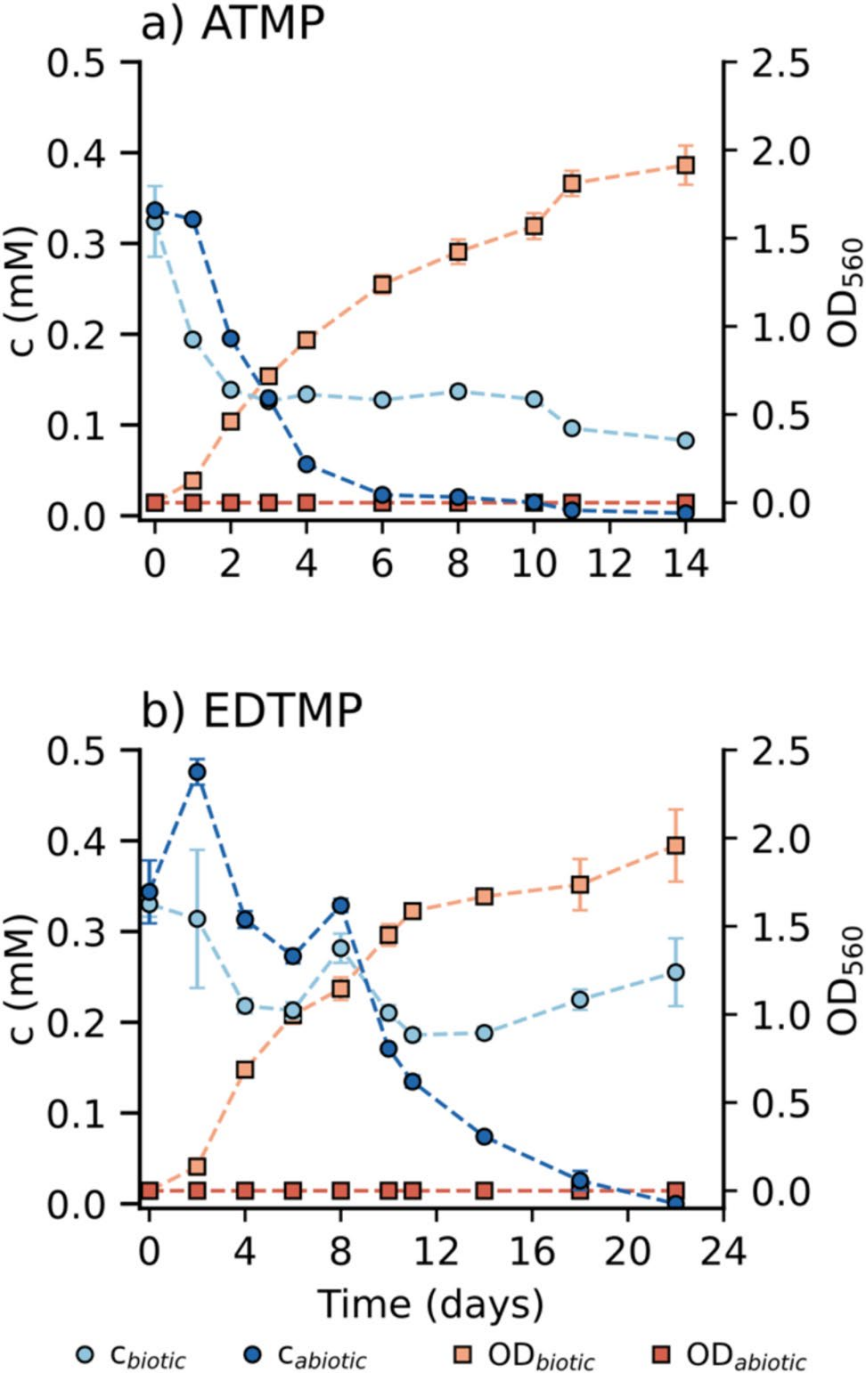
Changes in APP concentration and optical density of *A*. Kg 19 over time when **a** ATMP and **b** EDTMP were provided at 0.5 mM as sole P-sources to study the biotransformations potential of ATMP and EDTMP by strain *A*. Kg 19. Light-blue circles (connected by lines) represent the change in APP concentration in inoculated biotic setups, while dark-blue circles indicate APP concentrations in cell-free abiotic controls. Light-orange squares represent the optical density of *A*. Kg 19 growing cells with APP, and dark-orange squares show the optical density in abiotic setups. Error bars represent the standard deviation of triplicate cultures (if not visible, error bars are smaller than the symbols)

To investigate the biotransformation potential of higher APPs, ATMP and EDTMP were provided to strain *A*. Kg 19 as sole P-sources. The change in APP concentration, as well as the growth indicated by an increase in optical densities in biotic and abiotic setups, are shown in Fig. 1. Over time, a significant decrease in the ATMP and EDTMP concentrations was observed in biotic setups, accompanied by cell growth. The transformation of ATMP and EDTMP reached 74% and 22%, respectively, and plateaued after 4 to 6 days. A significant decrease in both APP concentrations, however, was also observed in cell-free abiotic controls (dark-blue data in Fig. 1), which led to the depletion of ATMP and EDTMP by days 6 and 18, respectively. Schowanek and Verstraete (1990) also reported abiotic degradation in the growth medium while investigating APP biodegradation. However, in their study, the biologically mediated degradation was up to two orders of magnitude faster than the abiotic process, and thus, the latter was not considered to significantly contribute to the total degradation of APPs. In contrast, the data shown in Fig. 1 demonstrate rapid abiotic breakdown of ATMP and EDTMP in the media matrix, occurring concurrently with potential biotransformation that cannot be neglected.

The cultivation medium used here has been frequently applied in previous studies to assess the biodegradability of aminomono- and diphosphonates such as glyphosate, aminomethylphosphonic acid (AMPA), IDMP, and 1-hydroxyethane-1,1-diphosphonate (HEDP) without significant reactivity in cell-free controls (Kourtaki et al. 2025). Yet, for higher APPs, the abiotic degradation shown in cell-free controls challenges the distinction between concurrent biotic and abiotic degradation of ATMP or EDTMP, hindering the assessment of strain *A*. Kg 19’s potential ability to use the tested APPs as sole P-sources.

### Effect of trace Mn(II) concentrations on ATMP

To further explore the abiotic degradation of higher APPs in sterile medium, follow-up experiments focused on ATMP, which exhibited greater susceptibility to degradation. Standard approaches to APP biodegradation are typically conducted using nutrient-rich growth media designed to support microbial activity. Riedel et al. (2023) proposed a standardized degradation test for assessing the biodegradability of higher APP and highly recommended the inclusion of a TES. These solutions typically contain metals such as Zn, Ca, Fe, Cu, Mn, and Ni, with dissolved Mn(II) added at concentrations between 2.5 and 10 µM (Ermakova et al. 2008; Riedel et al. 2023, 2024; Kourtaki et al. 2025). Notably, Mn(II) has been shown to catalyze abiotic oxidation of higher APPs by molecular oxygen in tap water and buffered distilled water (Nowack and Stone 2000).

**Fig. 2:**
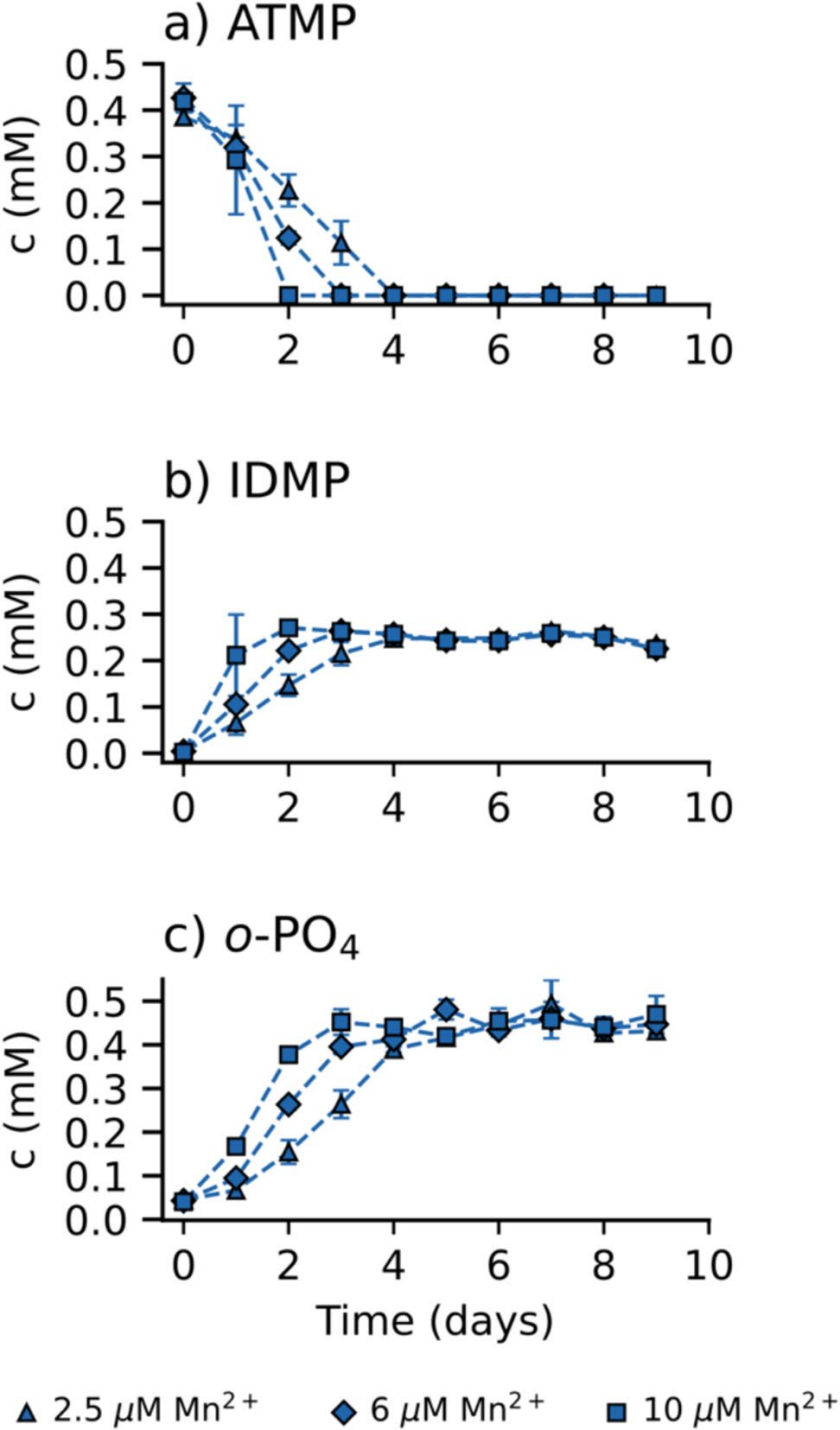
Investigation of ATMP (**a**), IDMP (**b**), and *o*-PO_4_ (**c**) concentrations vs time in the presence of various concentrations of Mn^2+^ in 50 mM MOPS buffer (pH 7.0). Triangles (connected by lines) indicate setups with 2.5 µM Mn^2+^ in 50 mM MOPS, diamonds (connected by lines) indicate setups with 6 µM Mn^2+^ in 50 mM MOPS, and squares (connected by lines) indicate setups with 10 µM Mn^2+^ in 50 mM MOPS. Error bars represent the standard deviation of triplicate setups (if not visible, error bars are smaller than the symbols)

In this study, we aimed to evaluate the role of dissolved Mn(II) present in the trace element solution with respect to abiotic ATMP degradation observed in the sterile growth medium. To this end, 0.5 mM ATMP in 50 mM MOPS buffer at pH 7.0 was supplemented with three different Mn(II) concentrations (2.5, 6, and 10 µM). The results of these experiments are shown in Fig. 2. A rapid decrease in ATMP concentration was observed across all tested Mn(II) concentrations. The ATMP degradation rate linearly correlated with Mn(II) concentration, with rates of 0.096 ± 0.003, 0.142 ± 0.010, and 0.210 ± 0.006 mM day^−1^ for Mn(II) levels of 2.5, 6, and 10 µM, respectively (*R*^2^ = 0.995, see Figure S2). ATMP degradation was accompanied by the formation of IDMP and *o*-PO_4_ as transformation products, confirming the occurrence of abiotic ATMP breakdown in the presence of trace Mn(II). While *o*-PO₄ formation was stoichiometric with the decrease of ATMP in all setups (Fig. 2c), IDMP was produced in sub-stoichiometric amounts (Fig. 2b). This suggests the involvement of alternative or multi-step degradation pathways that may yield additional, unidentified P-containing byproducts. This interpretation is supported by Marks et al. (2024), who investigated the photolytic degradation of ATMP and identified several P-containing transformation products beyond IDMP, including AMPA, hydroxymethylphosphonic acid (HMP), formylphosphonic acid, methylphosphonic acid, and phosphonic acid using high-resolution mass spectrometry. Interestingly, AMPA was not detected as a transformation product of IDMP in our experiments. Overall, these results clearly demonstrate that trace-concentrations of Mn(II) in bacterial growth media significantly compromise ATMP stability. This abiotic process, first described by Nowack and Stone (2000), involves a three-step oxidation mechanism: (1) the formation of an Mn(II)-ATMP complex, (2) oxygen-driven oxidation of Mn(II) to Mn(III) within the Mn(III)ATMP complex, and (3) intramolecular electron transfer leading to bond cleavage in ATMP and regeneration of Mn(II). Martin et al. (2022) further elucidated this process using stable carbon isotope analysis, identifying two parallel degradation pathways.

In summary, these results show that Mn(II), even at low micromolar concentrations – commonly used in biodegradation studies of higher APPs – can transform 0.5 mM ATMP into *o*-PO_4_, IDMP, and likely other P-containing degradation products. This has significant implications for biodegradation research, as the formation of such metabolites can confound the interpretation of microbial activity and lead to overestimation of true biodegradability.

### Effect of bacterial growth media composition on ATMP stability

**Fig. 3:**
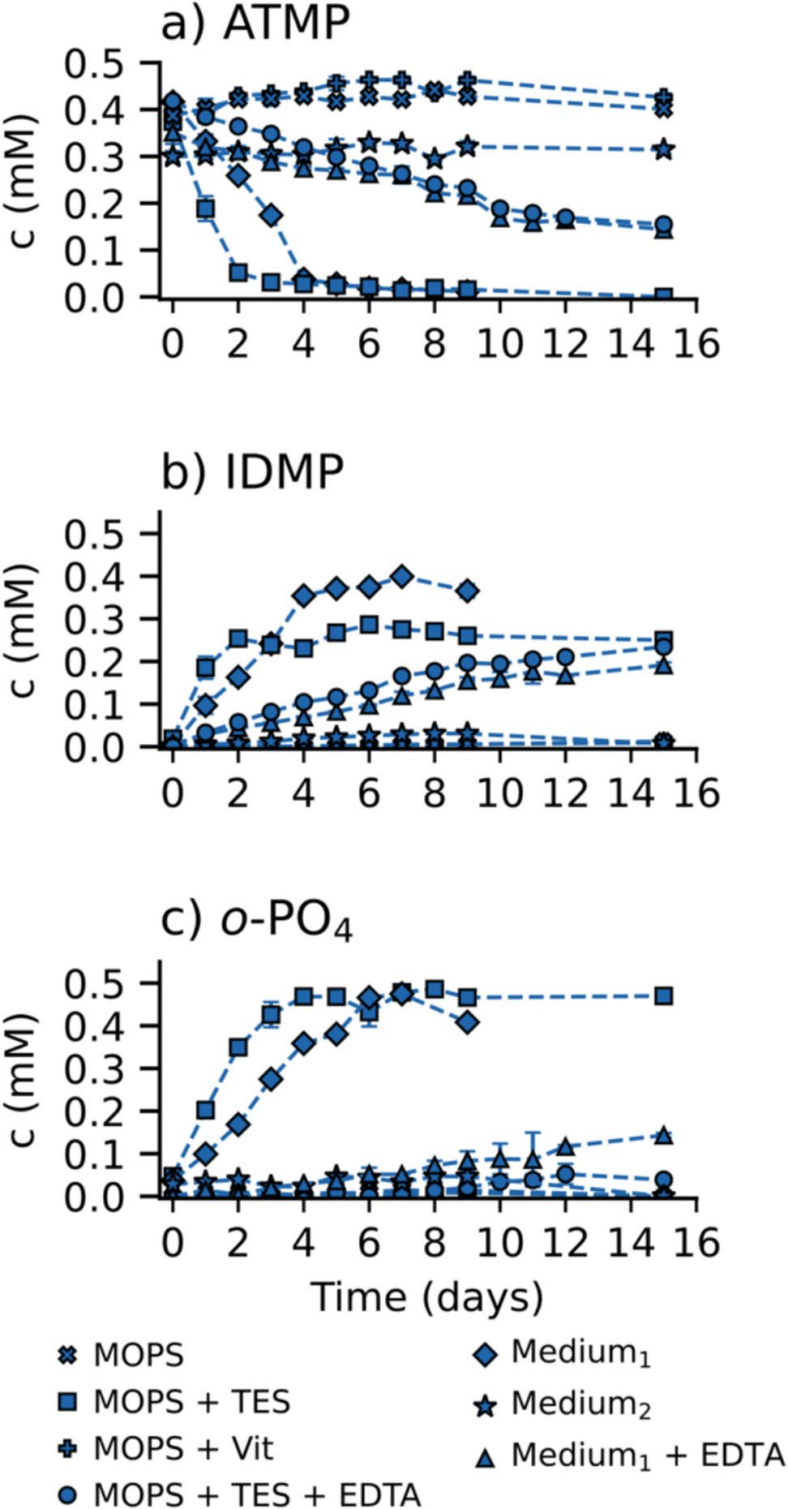
Investigation of ATMP (**a**), IDMP (**b**), and *o*-PO_4_ (**c**) concentrations vs time under various experimental conditions. Crosses (connected by lines) indicate setups with 50 mM MOPS, squares (connected by lines) indicate setups with 50 mM MOPS and TES, plus signs (connected by lines) indicate setups with 50 mM MOPS and vitamin solution, diamonds (connected by lines) describe the medim_1_ used for strain *A*. Kg 19, stars (connected by lines) describe the Medium_2_ for strain *O*. GPr1-13, triangles (connected by lines) describe the setups with Medium_1_ with 100 µm EDTA, and circles (connected by lines) demonstrate setups with 50 mM MOPS, trace element solution, and 100 µm EDTA. Error bars represent the standard deviation of triplicate setups (if not visible, error bars are smaller than the symbols)

To shed further light on the abiotic breakdown of ATMP in sterile growth medium, further experiments were conducted in seven different matrices (c_(ATMP)_ = 0.5 mM, pH = 7.0; preparation details in “Setup of abiotic transformation batch experiments” section), as summarized in Table 1. The concentrations of ATMP, IDMP, and *o*-PO_4_ in these setups are shown in Fig. 3. Consistent with previous results (Fig. 1), ATMP degraded rapidly in the medium used for cultivating *A*. Kg 19 (Medium_1_) (see Fig. 3a), with a corresponding release of IDMP and *o*-PO_4_ (Fig. 3a–c). Contrarily, ATMP remained stable in Medium_2_ over 9 days, with no release of IDMP or *o*-PO_4_ observed. Both media contained the same amounts of organic carbon and buffer but differed in the concentration of the nitrogen source (c_NH4Cl, Medium2_ = 2.5 × c_NH4Cl, Medium1_ ~ 93.5 mM) and the amount of Ca(II) (c_Ca2+, Medium2_ = 8 × c_Ca2+, Medium1_ ~ 365 µM). Most notably, Medium_2_ was not supplemented with a TES and consequently was manganese-free. The TES (Zn, Ca, Fe, Cu, Mn, and Ni) was identified as the driving factor for ATMP degradation in an experiment with TES-spiked MOPS. ATMP was completely degraded within 2 days (rate 0.161 mM day⁻^1^), producing stoichiometric amounts of *o*-PO₄ (0.47 mM) and sub-stoichiometric amounts of IDMP (0.26 mM). While the fastest degradation occurred in MOPS with the TES, ATMP also degraded in Medium_1_, albeit at a slower rate of 0.093 mM day⁻^1^. This reduced rate may be due to the presence of competing cations such as Mg(II) (~ 0.8 mM), which can competitively form complexes with ATMP, and potentially modulate the reaction kinetics. Nowack and Stone (2000) demonstrated that certain bivalent cations found in trace element solutions – such as Ca(II) and Zn(II) – do not promote ATMP degradation, but rather slow down the reaction by complex formation. This aligns with our observations: ATMP degraded more rapidly in MOPS with Mn(II) alone than in media also containing Cu(II) or Mg(II) (see Figs. 2 and 3). Interestingly, ATMP degradation also occurred in Medium_1_ amended with EDTA, a common metal chelator used in biodegradation studies (Forlani et al. 2011; Riedel et al. 2023, 2024). For 15 days, over 50% of ATMP was degraded, producing 0.19 mM IDMP and 0.14 mM *o*-PO_4_. Visual MINTEQ calculations showed that at the EDTA concentrations used, most metals-including Mn(II) were complexed (percentages equaled to 98.8%, 16.5%, 73.6%, 100%, 99.3%, and 0.2% for Fe(II), Ca(II), Cu(II), Zn(II), Mn(II), and Mg(II), respectively) (complex formation constants are summarized in Table S3). Despite this, metal-mediated ATMP degradation persisted, suggesting that even EDTA-complexed Mn(II) remains catalytically active. This was further confirmed in a simplified system containing only MOPS, trace elements, ATMP, and EDTA, where 63% degradation occurred by day 15. Thus, although EDTA significantly slowed the reaction, it did not fully inhibit it. Complementary tests with MOPS buffer, with or without a vitamin solution, showed no adverse effect on ATMP – no IDMP or *o*-PO₄ was released over the 9 days, indicating no hydrolysis.

Overall, in accordance with literature knowledge, our results clearly identify Mn(II) as the main driver of abiotic ATMP degradation in aqueous systems containing trace elements. Degradation was consistently accompanied by the release of IDMP and *o*-PO₄, confirming abiotic transformation under the tested conditions. Importantly, *o*-PO₄ is the most accessible P-source for microbes and can suppress the expression of phosphonate uptake and degradation genes at concentrations above 4 µM (Tetsch and Jung 2009; Stosiek and Klimek-ochab 2020). In mixed systems, these breakdown products themselves can serve as P-sources for microbial growth. Microorganisms are likely to first consume available *o*-PO₄ and smaller P-containing transformation products like IDMP before turning to the parent compound, such as ATMP.

These findings imply that previous studies on biodegradation of higher-APP in growth media containing EDTA and TES may have overlooked abiotic PO_4_^3−^ release driven by Mn(II)-driven oxidation. As a result, microbial growth observed in biodegradation experiments may reflect utilization of these more bioavailable metabolites rather than the parent compound. This undermines the use of growth as a proxy for biodegradation and may lead to significant overestimation of microbial degradation potential of higher APPs. Overall, these findings emphasize the need for careful distinction between biotic and abiotic processes when assessing the environmental fate of higher APPs.

## Conclusions and implications

This work demonstrates that common microbial growth media are inadequate for assessing the aerobic biodegradation of higher APPs such as ATMP and EDTMP due to the prevalence of the abiotic Mn(II)-catalyzed degradation. Trace concentration levels of dissolved Mn(II), commonly present in mineral media, were shown to rapidly transform ATMP, forming IDMP and *o*-PO_4_, even in the presence of the metal-chelating agent EDTA. Consequently, this abiotic process significantly interfered with the accurate assessment of ATMP and EDTMP biodegradability by *Achromobacter insolitus* strain Kg 19, as the parent compounds were rapidly transformed into other more bioavailable P-containing transformation products. The bacterial growth observed was likely supported by the utilization of such transformation products as P-source rather than by the direct biotransformation of ATMP or EDTMP. This suggests that microbial activity reported in previous studies may also have been sustained by such abiotic degradation intermediates, leading to a potential overestimation of the actual biodegradability of higher APPs. The release of *o*-PO₄ further complicates the interpretation of APP biodegradability by suppressing microbial APP utilization pathways, as observed previously in strain *A*. Kg 19.

While the degradation of higher APPs in the presence of oxygen and trace Mn(II) is a known environmental process, the role of Mn(II) has been largely overlooked in biodegradation studies of higher APPs. Thus, the findings presented here reveal a critical gap in current biodegradation testing approaches that fail to distinguish between microbially mediated degradation and metal-catalyzed abiotic reactions. Future research should focus on developing biodegradation approaches that will minimize abiotic interference while ensuring that media compositions support the full degradative potential of the test organisms. This includes the use of Mn(II)-free cultivation media (for microbes tolerant to Mn-deficient conditions) or media with bioavailable Mn-complexes, as well as the implementation of rigorous controls to clearly differentiate between biotic and abiotic transformation pathways. High-resolution mass spectrometry should also be employed to fully characterize degradation pathways and identify P-containing transformation products. This is particularly relevant for intermediates such as AMPA, which are environmentally persistent and ecologically concerning. Other products – such as methylphosphonic acid, phosphonic acid, and formylphosphonic acid—may also be formed during the degradation of higher APPs. These compounds can contribute to the overall P-load in the environment and may persist in soil and aquatic systems, influencing nutrient cycles and microbial communities. Such methodological improvements are essential for accurately assessing the biodegradability of higher APP – as non-degradable compounds can build up in ecosystems and pose long-term risks to both environmental and human health.

## Supplementary Information

Below is the link to the electronic supplementary material.

ESM1 (DOCX 149 KB)

## Data Availability

Data will be made available on request.
