# Supplementary material for: The overlooked role of manganese in biodegradation studies of higher aminopolyphosphonates
Source: Environ Sci Pollut Res Int. 2025 Nov 4;32(46):26602–10. doi: 10.1007/s11356-025-37105-9 (PMC12672635; doi:10.1007/s11356-025-37105-9)
Supplement: Supplementary file 1 — (DOCX 149 KB) [file 11356_2025_37105_MOESM1_ESM.docx]

**Supporting Information**

The Overlooked Role of Manganese in Biodegradation Studies
of Higher Aminopolyphosphonates

Kleanthi Kourtaki^1^, Philipp R. Martin^1,2^, Stefan B. Haderlein^1, *^

^1^Department of Geosciences, Eberhard Karls Universität Tübingen, Tübingen, GERMANY

^2^Division for Environmental Geosciences, Centre for Microbiology and Environmental Systems Science, University of Vienna, Austria

*Corresponding author: Stefan.haderlein@uni-tuebingen.de

Pages: 5

Figures: 2

Tables: 3

**2. Materials and methods**

*2.2 Setup of biodegradation batch experiments*

Biotransformation experiments were performed with a rod-shaped bacterial strain *Achromobacter insolitus* strain Kg 19 (hereafter will be referred to as *A*. Kg 19) isolated from the Krasnodar region in Russia (45°03’10.8’’N, 38°52’22.8’’E) and obtained as freeze-dried culture from the Russian Centre of Microorganisms (VKM) (Tarlachkov et al., 2020). Pre-cultures were maintained in a carbon – and nitrogen–rich medium, buffered with 50 mM MOPS and supplemented with glyphosate as the sole P-source. For each experiment, a fresh inoculum was prepared by cultivating *A*. Kg 19 for 5 to 6 days, until a dense culture was obtained (optical density (OD) >> 1). Each experiment was initiated with the addition of 100 µL of a cell suspension to 100 mL of medium. The inoculum was prepared on the day the experiment was started as follows: 5 mL of the freshly growing culture was harvested and was subjected to centrifugation for 10 minutes at 7,000 rcf. The resulting cell pellets were washed twice by resuspension in centrifugation tubes using each time 5 mL sterile P-free medium. After the second washing step, the cell pellets were resuspended in 2.5 mL of the P-free medium, from which 100 µL was withdrawn and used as inoculum. All experiments were standardized to begin with a similar starting cell number, resulting in a cell density of approximately 10^6^ cells mL^-1^. The medium composition for *A*. Kg 19 (Medium_1_) was comprised of: 10 (g/L) Na-glutamic acid as C-source, 2 (g/L) NH_4_Cl as N-source, with 0.5 mM of the respective APP as P-source and was buffered at pH 7±1 with 50 (mM) MOPS. Additionally, the medium was supplemented with 1 mL/L of a 7-Vitamin solution, 0.2 g/L MgSO_4_ x 7H_2_O, 0.5 (g/L) K_2_SO_4_, and a trace element solution with 2.5 (mg/L) FeSO_4_ x 7H_2_0, 10 (mg/L) CaCl_2_ x 6H_2_O, 2 (mg/L) CuSO_4_ x 5H_2_O, 0.06 (mg/L) H_3_BO_3_, 20 (mg/L) ZnSO_4_ x 7H_2_O, 1 (mg/L) MnSO_4_ x H_2_0, 0.05 (mg/L) NiCl_2_ x 6H_2_O, 0.3 (mg/L) Na_2_MoO_4_ x 2H_2_O. For all biotransformation experiments 100 mL glass serum bottles, sterilized by dry heat at 180°C for 4.5 hours we used. the bottles continuously shaken on a rotary shaker at 150 rpm. Each culture was prepared under sterile conditions, filled with 100 mL of sterile medium, and adjusted to the desired OP concentration by adding a sterile-filtered stock solution prepared in the respective media. The serum bottles were sealed with sterile, oxygen-permeable cotton stoppers. The experiment was initiated by inoculating bottles with the living cells. Each experiment consisted of three living replicates, and three abiotic controls to assess potential interactions with the APPs and the medium.

**Table S1.** Media composition for the cultivation of the *Achromobacter insolitus* strain Kg 19 (VKM B-3295) (hereafter will be referred to as *A*. Kg 19).

| **Compound** | **Empirical formula** | **Concentration** |
| --- | --- | --- |
| Monosodium glutamic acid (glutamate) | NaC_5_H_9_NO_4_ | 10 g L^-1^ |
| Ammonium chloride | NH_4_Cl | 2 g L^-1^ |
| Potassium sulfate | K_2_SO_4_ | 0.5 g L^-1^ |
| Magnesium sulfate heptahydrate | MgSO_4_ * 7H_2_O | 0.2 g L^-1^ |
| 3-(morpholino)propanesulfonic acid (MOPS) | C_7_H_15_NO_4_S | 50 mmol L^-1^ |
| Sodium hydroxide for pH adjustment | NaOH (stock 1 mol L^-1^) | - |
| 7-Vitamin solution |  | 1 mL L^-1^ |
| Zinc sulfate heptahydrate | ZnSO_4_ * 7H_2_O | 20 mg L^-1^ |
| Calcium chloride hexahydrate | CaCl_2_* 6H_2_O | 10 mg L^-1^ |
| Iron sulfate heptahydrate | FeSO_4_ * 7H_2_0 | 2.5 mg L^-1^ |
| Copper sulfate pentahydrate | CuSO_4_ * 5H_2_O | 2 mg L^-1^ |
| Manganese sulfate monohydrate | MnSO_4_ * H_2_O | 1 mg L^-1^ |
| Sodium molybdate | Na_2_MoO_4_ | 0.3 mg L^-1^ |
| Boric acid | H_3_BO_3_ | 0.06 mg L^-1^ |
| Nickel chloride hexahydrate | NiCl_2_ * 6H_2_O | 0.05 mg L^-1^ |

**Table S2.** Media composition for the cultivation of the *Ochrobactrum pituitosum*strain GPr1-13 (hereafter will be referred to as *O*. GPr1-13).

| **Compound** | **Empirical formula** | **Concentration** |
| --- | --- | --- |
| Monosodium glutamic acid (glutamate) | NaC_5_H_9_NO_4_ | 10 g L^-1^ |
| Ammonium chloride | NH_4_Cl | 5 g L^-1^ |
| Potassium sulfate | K_2_SO_4_ | 0.5 g L^-1^ |
| Magnesium sulfate heptahydrate | MgSO_4_ * 7H_2_O | 0.16 g L^-1^ |
| Calcium chloride dihydrate | CaCl_2_* 2H_2_O | 0.08 g L^-1^ |
| 3-(morpholino)propanesulfonic acid (MOPS) | C_7_H_15_NO_4_S | 50 mmol L^-1^ |
| Sodium hydroxide for pH adjustment | NaOH (stock 1 mol L^-1^) | - |
| 7-Vitamin solution |  | 1 mL L^-1^ |

**Figure S1:** pH-dependent speciation of ATMP. ATMP acidity constants for an ionic strength of 0.1 at 25°C taken from Deluchat et al., (1997).

**3. Results and Discussion**


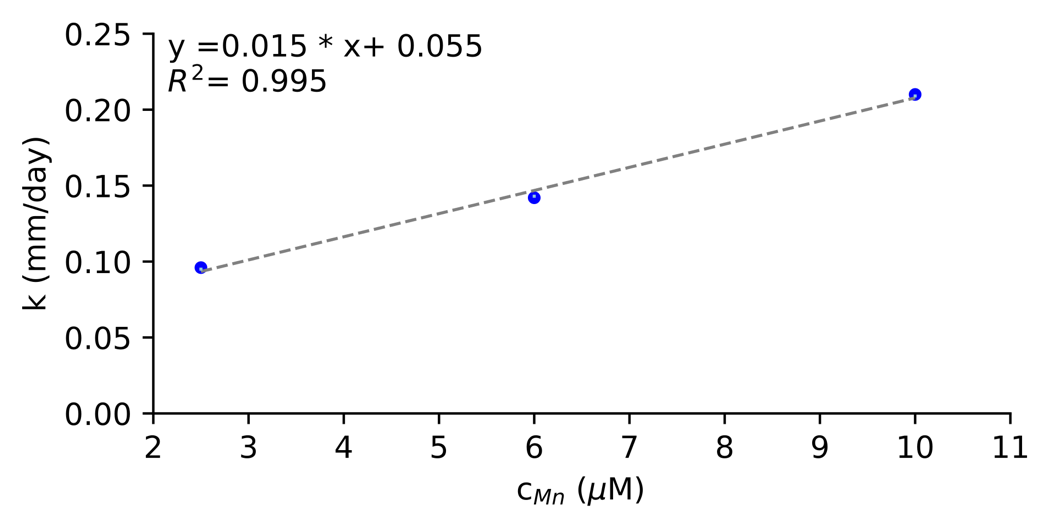


**Figure S2:** ATMP transformation rates as a function of Mn (II) concentration. The degradation rate increased linearly with Mn(II) levels, with measured rates of 0.096 ± 0.003, 0.142 ± 0.010, and 0.210 ± 0.006 mM day⁻¹ for Mn(II) concentrations of 2.5, 6, and 10 µM, respectively. Symbols represent the calculated rates, and the dashed line indicates the linear fit.

**Table S3**. Formation constants for metal-EDTA complexes extracted from thermodynamic database in VisualMINTEQ (thermo.vdb).

| **Ion** | **LogK** |
| --- | --- |
| Cu^2+^ | 20.5 |
| Zn^2+^ | 18.0 |
| Fe^2+^ | 16.0 |
| Mn^2+^ | 15.6 |
| Ca^2+^ | 12.4 |
| Mg^2+^ | 10.6 |

The formation of these complexes can be represented generally by the equilibrium:

$$M^{n}+{EDTA}^{-4}={MEDTA}^{n-4}$$

$$where:$$

$M^{n}=any metal cation$ (e.g., ${Cu}^{2+}, {Zn}^{2+}, {Fe}^{2+})$

$${MEDTA}^{n-4}=the metal-EDTA chelate complex$$

**REFERENCES**

Deluchat, V., Bollinger, J. C., Serpaud, B., & Caullet, C. (1997). Divalent cations speciation with three phosphonate ligands in the pH-range of natural waters. *Talanta*, *44*(5), 897–907. https://doi.org/10.1016/S0039-9140(96)02136-4

Tarlachkov, S. V., Epiktetov, D. O., Sviridov, A. V., Shushkova, T. V., Ermakova, I. T., & Leontievsky, A. A. (2020). Draft Genome Sequence of Glyphosate-Degrading Achromobacter insolitus Strain Kg 19 (VKM B-3295), Isolated from Agricultural Soil. *Microbiology Resource Announcements*, *9*(17), 19–20. https://doi.org/10.1128/mra.00284-20
